# Bridging Thrombolysis Versus Direct Endovascular Thrombectomy in Acute Ischemic Stroke Patients With ASPECTS ≤5: A Meta‐Analysis

**DOI:** 10.1002/brb3.71851

**Published:** 2026-10-22

**Authors:** Shuang Wang, Bing Wu, Fang Liu, Guiyan Sun

**Affiliations:** ^1^ Department of Neurology Army 78th Military Group Hospital Mudanjiang China

**Keywords:** Acute ischemic stroke, ASPECTS, Bridging thrombolysis, Endovascular thrombectomy, Intravenous thrombolysis

## Abstract

**Background:**

Endovascular thrombectomy (EVT) is a standard treatment for acute ischemic stroke (AIS) caused by large vessel occlusion (LVO). However, the efficacy and safety of bridging thrombolysis (BT) compared to direct EVT (dEVT) remain controversial, particularly in patients with large core infarcts (Alberta stroke program early CT score [ASPECTS] ≤5). This meta‐analysis aimed to compare the efficacy and safety of BT versus dEVT in this population.

**Methods:**

We systematically searched PubMed, Embase, and Web of Science from January 1, 2010, to September 21, 2024, for studies comparing BT and dEVT in AIS patients with ASPECTS ≤5. Outcomes included functional outcomes (mRS 0–2 and 0–3), intracranial hemorrhage (ICH), symptomatic ICH (sICH), 90‐day mortality, and successful reperfusion. Data were pooled using random‐effects models.

**Results:**

Twelve studies (3907 patients) were included. BT significantly improved favorable (OR: 1.43, 95% CI: 1.17–1.76) and moderate functional outcomes (OR: 1.28, 95% CI: 1.10–1.48) compared to dEVT. No significant differences were observed in sICH, ICH, 90‐day mortality, or successful reperfusion.

**Conclusions:**

Our findings suggest that BT may be associated with better functional outcomes in patients with ASPECTS ≤5, without increasing major safety events compared with dEVT. However, these findings should be interpreted cautiously because the evidence is mainly observational, and residual confounding cannot be excluded. Further well‐designed randomized controlled trials are needed to confirm these findings.

## Graphical abstract

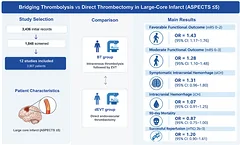

## 1 Introduction

Endovascular thrombectomy (EVT) has become a well‐established treatment for acute ischemic stroke (AIS) caused by anterior circulation large‐vessel occlusion (LVO), providing significant clinical benefit in improving patient outcomes (Powers et al. 2019; Turc et al. 2019). However, there is ongoing debate regarding whether to direct endovascular thrombectomy (dEVT) or to combine it with intravenous thrombolysis (IVT) before the thrombectomy (Turc et al. 2022). A recent meta‐analysis of individual data suggests no difference between bridging thrombolysis (BT) and dEVT in the treatment of AIS due to LVO (Majoie et al. 2023).

Early EVT trials often excluded patients with large ischemic core infarcts due to concerns about poor outcomes and increased risk of hemorrhagic transformation. However, recent six randomized controlled trials (RCTs) have demonstrated that EVT significantly improves outcomes for patients with large‐core ischemic strokes when compared to traditional medical management alone (Yoo et al. 2024; Bendszus et al. 2023; Costalat et al. 2023; Sarraj et al. 2023; Yoshimura et al. 2022; Huo et al. 2023). Nevertheless, data on the efficacy and safety of dEVT compared with BT in large‐core ischemic stroke remain limited and inconsistent (Shindo et al. 2024; Yuan et al. 2024; Derraz et al. 2023).

Although three previous meta‐analyses have attempted to address this issue, the substantial differences in inclusion criteria and conclusions across studies make the results difficult to generalize (Guo et al. 2025; Wang et al. 2024; Liu et al. 2024). Some studies favored BT, citing better recanalization rates and functional outcomes (Guo et al. 2025; Liu et al. 2024), while others suggested BT exhibited similar functional and safety outcomes compared to EVT alone (Wang et al. 2024). The major limitations of these meta‐analyses include inconsistent definitions of large core infarct, variability in study designs, and inconsistent numbers of included studies. Given the inconclusive results from previous meta‐analyses, this study aims to conduct a comprehensive meta‐analysis comparing BT and dEVT in patients with large‐core infarcts, using stricter inclusion criteria to clarify the optimal treatment strategy.

## 2 Methods

This systematic review and meta‐analysis adhered to the preferred reporting items for systematic reviews and meta‐analyses (PRISMA) guidelines and was registered in the PROSPERO database (CRD42024593074).

### 2.1 Data Sources and Search Strategy

We systematically searched PubMed, Embase, and Web of Science databases from January 1, 2010, to September 21, 2024, to identify studies comparing BT and dEVT in AIS patients with an Alberta stroke program early CT score (ASPECTS) score of ≤5. The search strategy combined terms related to stroke, thrombectomy, intravenous thrombolysis, and large core infarcts (e.g., “stroke,” “thrombectomy,” “intravenous thrombolysis,” “large core,” “ASPECTS”). The detailed search strategies are provided in Table S1. The language was restricted to English. In addition, we manually screened reference lists from relevant articles to ensure comprehensive literature coverage.

### 2.2 Inclusion and Exclusion Criteria

Studies were included if they met the following criteria: (1) cohort studies that compared BT and dEVT in patients with AIS due to large core infarcts; (2) large core infarcts were defined as having an ASPECTS score of ≤5 on noncontrast CT (NCCT) or diffusion‐weighted MRI (DW‐MRI); (3) the sample size ≥50; (4) studies reporting outcomes such as favorable functional outcome (FFO) (modified Rankin Scale [mRS] 0–2 at 90 days), moderate functional outcome (MFO) (mRS 0–3 at 90 days), any intracranial hemorrhage (ICH), symptomatic intracranial hemorrhage (sICH), all‐cause mortality at 90 days, and successful reperfusion (SR) defined as modified thrombolysis in cerebral infarction (mTICI) 2b–3; and (5) availability of full‐text articles.

The exclusion criteria were as follows. (1) Studies that did not report the ASPECTS score; (2) studies using perfusion imaging alone to define large ischemic core (e.g., ≥50 or 70 mL) without ASPECTS ≤5; (3) case reports, reviews, conference abstracts, or studies that lacked a control group; and (4) duplicate publications (the largest sample size was included).

### 2.3 Data Extraction and Outcome Measures

Two reviewers (B.W. and F.L.) independently reviewed the titles and abstracts of the retrieved articles, excluding those that did not meet the eligibility criteria. Subsequently, they screened the full texts of potentially relevant articles and extracted the corresponding data into a structured dataset. The following variables were collected from each eligible study: first author, year of publication, study country, study duration, study design, sample size, number of males, age, occlusion location, study time window, baseline NIHSS score, definition of large core, imaging modality, number of mTICI 2b–3 after EVT, study outcome, definition of sICH, and quality of research.

The primary outcome was FFO. Secondary outcomes included MFO, 90‐day all‐cause mortality, sICH, ICH, and SR. Discrepancies between the reviewers were resolved by discussion and consensus, with consultation from a third reviewer (G.S.).

### 2.4 Risk of Bias and Quality Assessment

The quality of the studies was assessed by the Newcastle–Ottawa Scale. High quality was defined as a grade >6. Two authors (B.W. and F.L.) independently performed the risk of bias assessment, and any disagreements were resolved through discussion with a third reviewer (G.S.).

### 2.5 Statistical Analysis

Odds ratios (ORs) and risk differences (RDs) with 95% confidence intervals (CIs) were calculated for dichotomous outcomes. The DerSimonian–Laird method with random‐effects models was used due to the anticipated heterogeneity between studies. Statistical heterogeneity was assessed using the *I* ^2^ statistic, where values of 25%, 50%, and 75% indicated low, moderate, and high heterogeneity, respectively. Publication bias was evaluated using Egger's test (*n* ≥10). Sensitivity analyses were conducted for each outcome by excluding each study included to test the robustness of the results (*n* ≥10). Symptomatic ICH was further analyzed in subgroups according to different definitions used across studies. A *p*‐value of less than 0.05 was considered statistically significant. We conducted the meta‐analysis using Stata/SE (version 17.0).

## 3 Results

### 3.1 Study Selection and Characteristics

Systematic search of databases initially yielded 3436 records. After removing 1588 duplicates, 1848 records were screened. Title and abstract screening identified 27 reports for full‐text assessment. During the assessment, 19 studies were excluded for various reasons. An additional 18 reports were identified through manual searches, with 4 ultimately meeting the inclusion criteria. A total of 12 studies were included in this systematic review and meta‐analysis (Shindo et al. 2024; Yuan et al. 2024; Derraz et al. 2023; Sun et al. 2025; Alexandre et al. 2024; Almallouhi et al. 2024; Gu et al. 2023; Broocks et al. 2023; Jia et al. 2022; Lei et al. 2021; Kaesmacher et al. 2021; Manceau et al. 2018). The study selection flowchart is presented in Figure 1.

**FIGURE 1:**
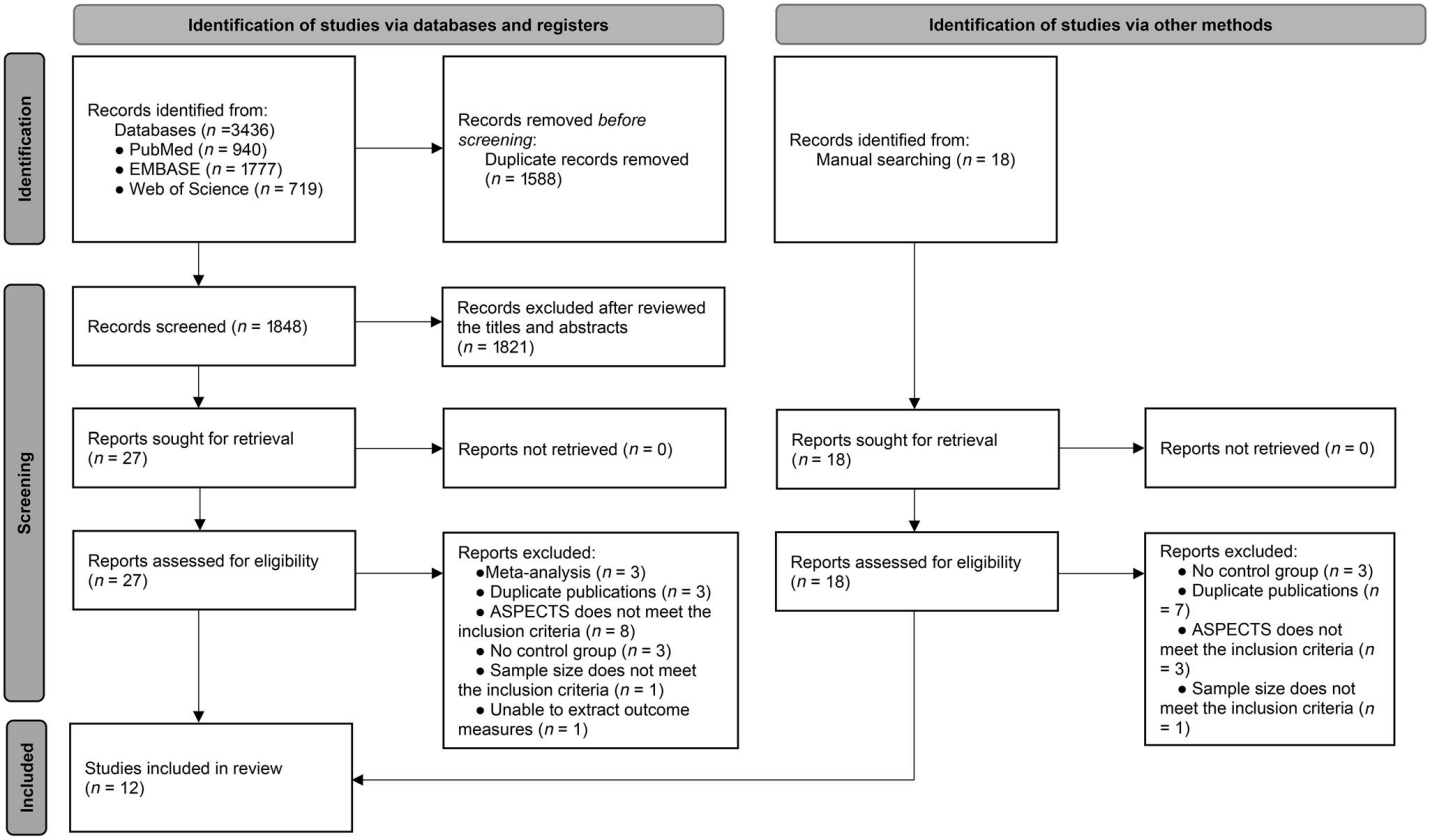
Flow diagram of the study search and selection process.

Table 1 summarizes the characteristics and quality assessments of the included studies. The studies were published between 2017 and 2024. Among the 3907 patients, 278 (7.1%) were from post‐hoc RCT analyses (3 studies), and 3629 (92.9%) from observational cohorts (9 studies). Sample sizes vary widely, with dEVT groups ranging from 25 to 754 patients and BT groups from 26 to 654. Most studies reported high mTICI 2b–3 rates in both dEVT and BT groups. Quality scores ranged from 6 to 8, indicating generally robust methodological quality (Table S2).

**TABLE 1:** Basic characteristics of the studies included in the meta‐analysis.

ID	Author	Publication year	Country	Duration	Study design	Sample size	Male (%)	Age, y	Occlusion location	Time window of EVT	Baseline NIHSS	Large core definition	Imaging modality	EVT mTICI (2b–3) (%)	Studyoutcome	sICH definition	Quality of research
1	Sun et al. (2025)	2024	China	2021– 2023	P (M)	dEVT: 368 BT: 122	dEVT: 209 (56.8) BT: 70 (59.0)	dEVT: 69 (58–78) BT: 69 (61–77)	ICA M1 M2	< 24 h	dEVT: 17 (14–21) BT: 17 (14–20)	ASPECTS ≤5	NCCT	dEVT: 316 (85.9) BT: 107 (87.7)	FFO; MFO; sICH; ICH; Mortality	HBC	8
2	Yuan et al. (2024)	2024	China	2020– 2022	PHA (RCT)	dEVT: 77 BT: 45	dEVT: 39 (50.6) BT: 27 (60.0)	dEVT: 67.5 ± 7.7 BT: 67.1 ± 7.8	ICA M1 M2	< 24 h	dEVT: 17 (14–20) BT: 18 (13–20)	ASPECTS (3–5) ASPECTS (0–2) and core (70–100 mL) ASPECTS (> 5) and core (70–100 mL)	NCCT	dEVT: 60 (90.9) BT: 32 (86.5)	FFO; MFO; sICH; ICH; Mortality	HBC	8
3	Alexandre et al. (2024)	2023	Italy France	2016– 2021	R (M)	dEVT: 265 BT: 143	NR	NR	ICA M1 M2 Tandem occlusion	NR	NR	ASPECTS ≤5	NCCT CTA	dEVT: 219 (82.6) BT: 112 (78.3)	FFO; MFO;	NR	7
4	Almallouhi et al. (2024)	2023	USA	2016– 2022	R (M)	dEVT: 355 BT: 202	NR	NR	ICA M1 M2 Tandem occlusion	< 24 h	NR	ASPECTS ≤5	NCCT	NR	FFO; MFO; sICH; ICH; Mortality	SITS‐MOST	6
5	Shindo et al. (2024)	2023	Japan	2018– 2021	PHA (RCT)	dEVT: 74 BT: 26	NR	NR	ICA M1 M2 Tandem occlusion	< 24 h	NR	ASPECTS (3–5)	NCCT MRI	dEVT: 65 (87.8) BT: 21 (80.8)	FFO; MFO; sICH; ICH; Mortality	SITS‐MOST	8
6	Derraz et al. (2023)	2023	France	2015– 2022	R (M)	dEVT: 754 BT: 654	dEVT: 416 (55.2) BT: 373 (57.0)	dEVT: 68.3 ± 15.4 BT: 67.1 ± 15.4	ICA M1 M2 Tandem occlusion	NR	dEVT: 19 (16–23) BT: 19 (16–22)	ASPECTS ≤5	NCCT MRI	dEVT: 590 (78.2) BT: 552 (84.4)	FFO; MFO; sICH; ICH; Mortality	ECASS II	8
7	Gu et al. (2023)	2023	China	2019– 2021	R (S)	dEVT: 32 BT: 27	NR	NR	ICA M1 M2 Tandem occlusion	< 6 h	NR	ASPECTS ≤5	NCCT	NR	FFO	NR	6
8	Broocks et al. (2023)	2022	Germany	2015– 2018	R (M)	dEVT: 78 BT: 90	NR	NR	ICA M1 M2	NR	NR	ASPECTS ≤5	NCCT	dEVT: 53 (67.9) BT: 68 (75.5)	FFO; MFO; sICH; Mortality;	ECASS II	7
9	Jia et al. (2022)	2022	China	2015– 2019	PHA (RCT)	dEVT: 25 BT: 31	dEVT: 13 (52.0) BT: 14 (45.1)	dEVT: 71 (68–78) BT: 66 (59–75)	ICA M1 M2	< 4.5 h	dEVT: 20 (15–23) BT: 20 (15–25)	ASPECTS ≤4	NCCT	dEVT: 20 (67.9) BT: 29 (75.5)	FFO; MFO; sICH; Mortality	HBC	6
10	Lei et al. (2021)	2021	China	2016– 2019	R (S)	dEVT: 108 BT:44	NR	NR	ICA MCA Tandem occlusion	< 6 h	NR	ASPECTS ≤5	NR	NR	FFO; Mortality	NR	7
11	Kaesmacher et al. (2021)	2021	Switzerland Canada Germany Spain France	2009– 2020	R (M)	dEVT: 178 BT:127	NR	NR	ICA M1 M2	NR	NR	ASPECTS ≤5	NCCT MRI	NR	FFO; sICH; Mortality	ECASS II	7
12	Manceau et al. (2018)	2017	France	2010– 2016	R (S)	dEVT: 28 BT:54	NR	NR	ICA MCA Tandem occlusion	< 6 h	NR	ASPECTS ≤5	MRI	NR	FFO	NR	6

Abbreviations: ACA, anterior cerebral artery; ASPECTS, Alberta stroke program early CT score; BT, bridging thrombolysis; CTA, CT angiography; dEVT, direct endovascular thrombectomy; ECASS, European Cooperative Acute Stroke Studies; FFO, poor functional outcome; HBC, Heidelberg bleeding classification; ICA, internal carotid artery; ICH, intracranial hemorrhage; M, multi‐center; M1, M1 segment of the middle cerebral artery; M2, M2 segment of the middle cerebral artery; MCA, middle cerebral artery; MFO, moderate functional outcome; MRI, magnetic resonance imaging; mRS, modified Rankin scale; mTICI, modified Thrombolysis in cerebral infarction; NCCT, noncontrast computed tomography; NIHSS, National Institute of Health Stroke Scale; NR, not report; P, prospective cohort; PHA, post hoc analysis; R, retrospective cohort; RCT, randomized, controlled trial; S, single‐center; sICH, symptomatic intracranial hemorrhage; SITS‐MOST, safe implementation of thrombolysis in stroke‐monitoring study.

### 3.2 Favorable Functional Outcome

A meta‐analysis of 12 studies showed a significantly higher likelihood of FFO in the BT group than dEVT. The OR was 1.43 (95% CI: 1.17–1.76, *p < *0.05, *I* ^2^ = 26.78%) (Figure 2) and the RD was 6.0% (95% CI: 2.0–10.0%, *p < *0.05, *I* ^2^ = 36.12%) (Figure S1). No publication bias was detected (Egger's test, *p =  *0.6601). Sensitivity analysis, performed by sequentially excluding each study, confirmed the robustness of the findings, demonstrating consistent results across all analyses (Figure S7).

**FIGURE 2:**
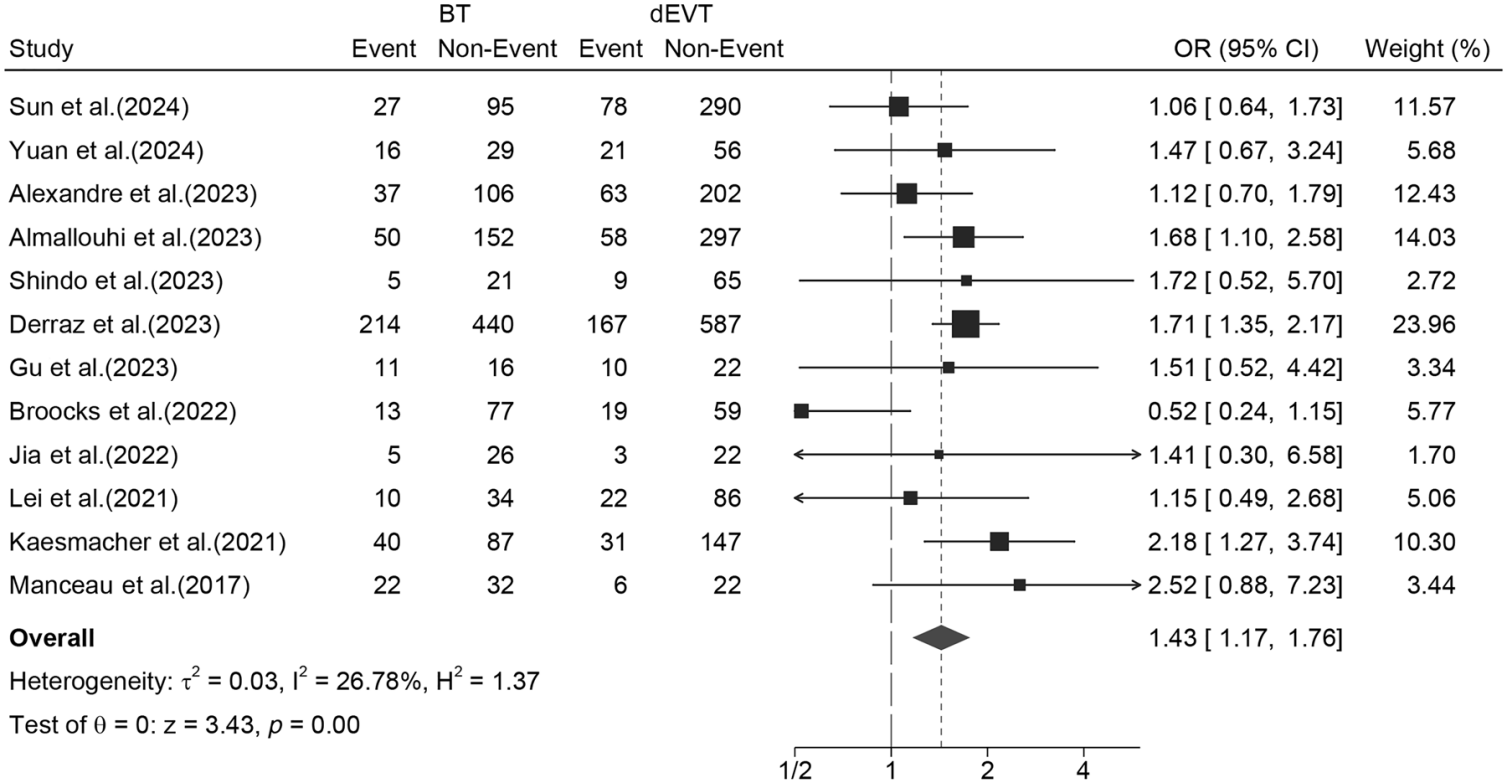
Forest plot for favorable functional outcomes (mRS 0–2 at 90 days) in patients receiving bridging therapy versus direct endovascular thrombectomy alone. Data from 12 studies (n = 3907 patients); odds ratio (OR) 1.43 (95% CI: 1.17–1.76, *p* < 0.05, *I* ^2^ = 26.78%); favors bridging therapy; no publication bias detected (Egger's test, *p = * 0.6601).

### 3.3 Moderate Functional Outcome

A meta‐analysis of 8 studies demonstrated a significantly higher likelihood of MFO in the BT group compared with dEVT. The OR was 1.28 (95% CI: 1.10–1.48, *p < *0.01, *I* ^2^ = 0.00%), and the RD was 5.0% (95% CI: 1.0–9.0%, *p = *0.01, *I* ^2^ = 6.46%) (Figure 3 and Figure S2).

**FIGURE 3:**
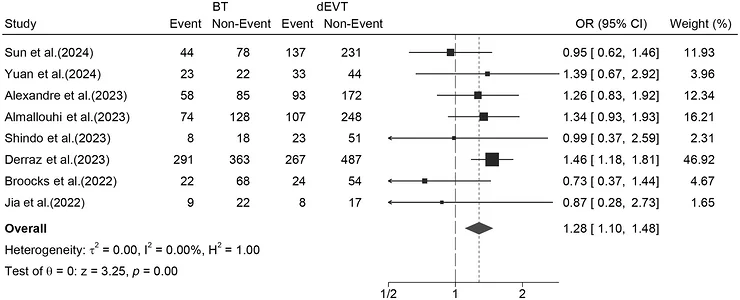
Forest plot for moderate functional outcome (mRS 0–3 at 90 days) in patients receiving bridging therapy versus direct endovascular thrombectomy alone. Data from 8 studies; OR 1.28 (95% CI: 1.10–1.48, *p* < 0.01, *I* ^2^ = 0.00%); favors bridging therapy.

### 3.4 Symptomatic Intracranial Hemorrhage

A meta‐analysis of 8 studies assessing sICH showed no statistically significant difference between the BT and dEVT groups. The OR for sICH was 1.31 (95% CI: 0.96–1.80, *p = *0.09, I^2^ = 29.16%), and the RD was 3.0% (95% CI: ‐1.0–6.0%, *p = *0.10, I^2^ = 33.29%) (Figure 4 and Figure S3). Further subgroup analyses based on sICH definitions showed an OR of 1.63 (95% CI: 0.89–2.99, *p = *0.08) for the European Cooperative Acute Stroke Study II (ECASS II), 0.75 (95% CI: 0.41–1.36, *p = *0.54) for the Heidelberg Bleeding Classification (HBC), and 1.58 (95% CI: 0.97–2.58, *p = *0.92) for the safe implementation of treatments in stroke‐monitoring study (SITS‐MOST), all consistent with the overall result.

**FIGURE 4:**
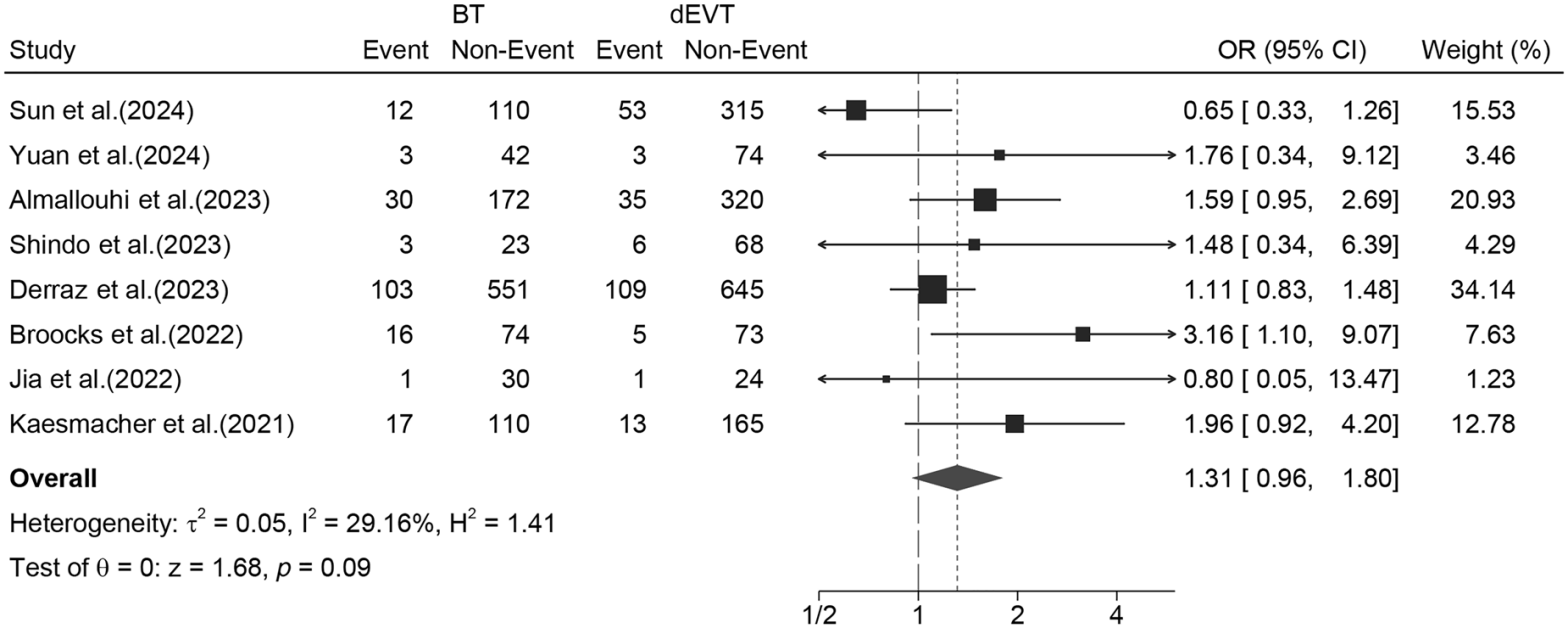
Forest plot for symptomatic intracranial hemorrhage in patients receiving bridging therapy versus direct endovascular thrombectomy alone. Data from 8 studies; OR 1.31 (95% CI: 0.96–1.80, *p = *0.09, *I* ^2^ = 29.16%); no significant difference.

### 3.5 Intracranial Hemorrhage

A meta‐analysis of 6 studies evaluating overall ICH showed no significant difference between the BT and dEVT groups. The OR was 1.07 (95% CI: 0.91–1.25, *p = *0.44, *I* ^2^ = 0.00%), and the RD was 2.0% (95% CI: ‐2.0–5.0%, *p = * 0.44, *I* ^2^ = 0.00%) (Figure 5 and Figure S4).

**FIGURE 5:**
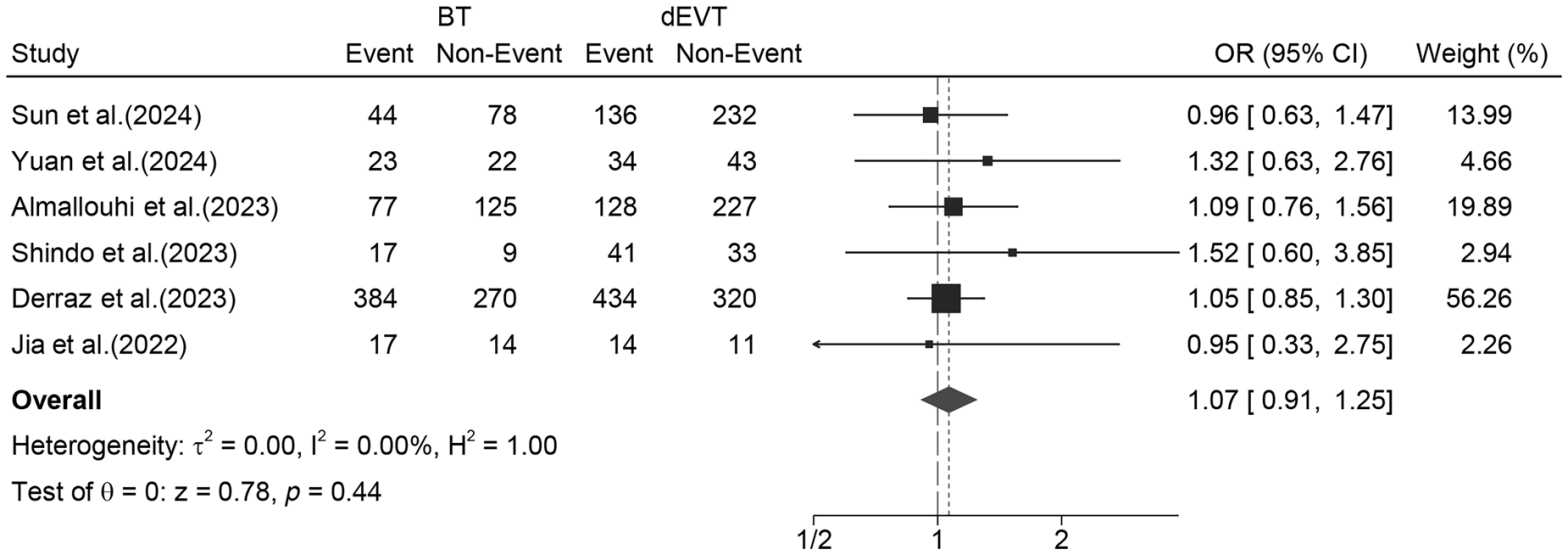
Forest plot for intracranial hemorrhage in patients receiving bridging therapy versus direct endovascular thrombectomy alone. Data from 6 studies; OR 1.07 (95% CI: 0.91–1.25, *p = *0.44, I^2^ = 0.00%); no significant difference.

### 3.6 Mortality at 90 Days

A meta‐analysis of 9 studies on 90‐day mortality showed no statistically significant difference between the BT and dEVT groups. The OR for mortality was 0.87 (95% CI: 0.75–1.00, *p = *0.06, *I* ^2^ = 0.00%), and the RD was −3.0% (95% CI: ‐6.0%–0.1%, *p = * 0.06, *I* ^2^ = 0.00%) (Figure 6 and Figure S5).

**FIGURE 6:**
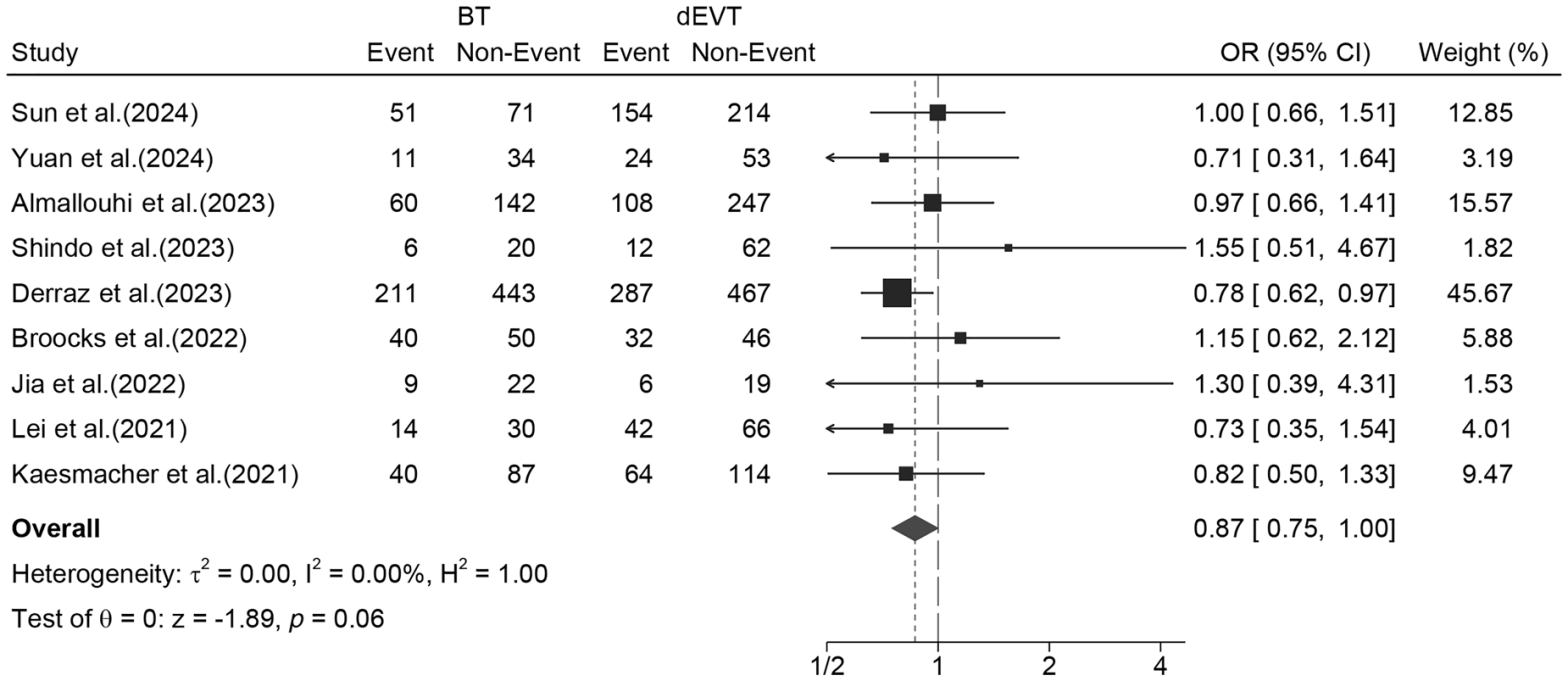
Forest plot for mortality at 90 days in patients receiving bridging therapy versus direct endovascular thrombectomy alone. Data from 9 studies; OR 0.87 (95% CI: 0.75–1.00, *p = *0.06, *I* ^2^ = 0.00%); no significant difference.

### 3.7 Successful Reperfusion

A meta‐analysis of 8 studies evaluating SR showed no significant difference between the BT and dEVT groups. The OR for achieving SR was 1.20 (95% CI: 0.90–1.61, *p = * 0.22, *I* ^2^ = 40.75%), and the RD was 3.0% (95% CI: −1.0%–7.0%, *p = *0.14, *I* ^2^ = 38.56%) (Figures 7 and Figure S6).

**FIGURE 7:**
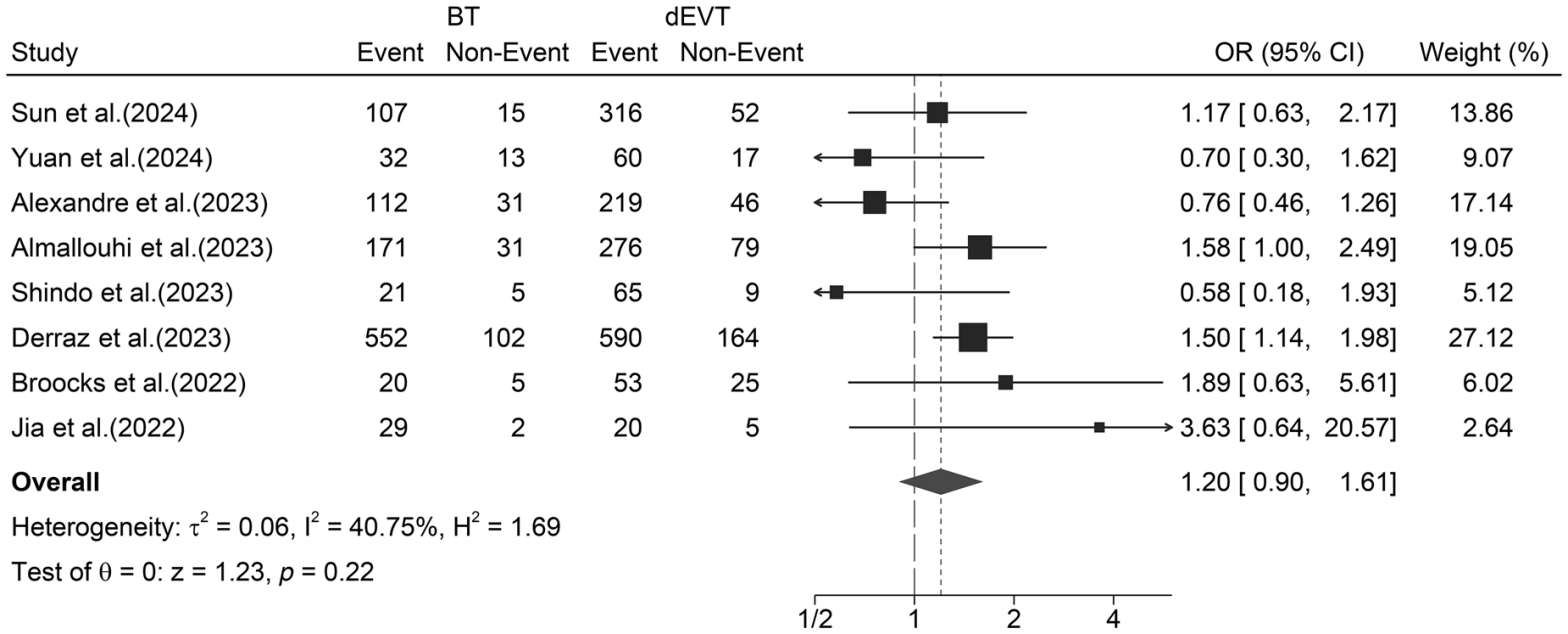
Forest plot for successful reperfusion (mTICI 2b–3) in patients receiving bridging therapy versus direct endovascular thrombectomy alone. Data from 8 studies; OR 1.20 (95% CI: 0.90–1.61, *p = *0.22, *I* ^2^ = 40.75%); no significant difference.

## 4 Discussion

This meta‐analysis aimed to compare the efficacy and safety of BT and dEVT in patients with large ischemic core infarcts, defined by an ASPECTS score of ≤5. Our findings suggest that BT may be associated with improved functional outcomes at 90 days compared to dEVT, without a statistically significant increase in sICH, ICH, or 90‐day mortality. No significant differences were observed in SR rates between the two groups. Nevertheless, these findings should be interpreted cautiously because most included studies were observational, and the pooled analyses were based on study‐level data.

The comparative value of BT and dEVT in anterior‐circulation LVO stroke remains controversial, particularly in patients with large infarct cores. Previous RCTs in the general LVO population have yielded inconsistent results, and a prior meta‐analysis did not confirm the non‐inferiority of dEVT to BT (Majoie et al. 2023; Yang et al. 2020; Zi et al. 2021; Suzuki et al. 2021; LeCouffe et al. 2021; Mitchell et al. 2022; Fischer et al. 2022). In patients with ASPECTS ≤5, the evidence has been more limited because previous subgroup analyses included only a small number of patients. Notably, observational data from the European SITS‐ISTR study suggested better functional independence and lower 3‐month mortality with BT (Ahmed et al. 2021), while post hoc analyses from the RESCUE‐Japan LIMIT and ANGEL‐ASPECT trials provided additional comparative evidence in this subgroup (Shindo et al. 2024; Yuan et al. 2024). By incorporating the latest cohort studies and post hoc analyses, our study expands the current evidence base in 3907 patients with ASPECTS ≤5 and suggests that BT may be associated with improved 90‐day functional outcomes without a statistically significant increase in safety risks.

Our findings are consistent with previous meta‐analyses showing improved functional outcomes with BT compared with dEVT alone (Guo et al. 2025; Liu et al. 2024). Importantly, the present study extends these findings by focusing specifically on patients with ASPECTS ≤5, a subgroup in whom treatment decisions are particularly challenging. Administering IVT before EVT can promote early reperfusion, shorten procedural time, dissolve distal emboli, and improve microvascular reperfusion (Chalos et al. 2019; Di Maria et al. 2018), thereby contributing to the improved functional recovery observed in the BT group.

Reperfusion success is influenced by a variety of factors, including etiologic subtype, collateral grading, thrombosis size, first‐line thrombectomy technique, and first‐pass effect (Chen et al. 2024). In our analysis, no significant difference in SR rates was observed between BT and dEVT, which is consistent with previous studies (Guo et al. 2025; Wang et al. 2024). However, despite similar SR rates, patients in the BT group had better functional outcomes. In patients with large‐core infarction, greater thrombus burden, more extensive vascular injury, and proximal occlusion may reduce the effectiveness of thrombolysis and limit improvements in SR (Ahmed et al. 2021). Nevertheless, BT may still facilitate the clearance of distal microemboli and improve microcirculatory perfusion, collateral flow, and tissue‐level reperfusion, thereby contributing to better neurological recovery (Faizy et al. 2023). Alternatively, this apparent dissociation between SR and functional outcomes may also reflect residual confounding, including differences in IVT eligibility, baseline stroke severity, or center‐level treatment expertise.

Regarding safety, our analysis found no significant increase in hemorrhagic complications, including sICH and ICH, with BT compared with dEVT. These findings are consistent with Liu et al. (2024) and Wang et al. (2024), who also observed no increased risk of hemorrhagic events associated with BT. In contrast, Guo et al. (2025) reported a higher risk of sICH with BT, which may be partly explained by differences in the definition of sICH, as their analysis did not stratify results according to hemorrhage criteria. Although IVT has theoretical hemorrhagic risks, such as blood–brain barrier disruption and clot fragmentation or displacement (Chalos et al. 2019; Di Maria et al. 2018), our findings indicate that BT does not increase the risk of adverse safety events, even in patients with large ischemic core infarcts. Furthermore, no significant difference in 90‐day mortality was observed between the two groups, further supporting the safety of BT in this population.

This meta‐analysis has several strengths. First, it includes a large and diverse cohort (3907 patients) across 12 studies, improving the statistical power to detect treatment differences. Second, our meta‐analysis focused on anterior circulation LVO patients with large infarct cores (ASPECTS ≤5 on NCCT or DW‐MRI), thereby reducing heterogeneity arising from variable imaging definitions by excluding patients with ASPECTS >5 and perfusion‐defined ischemic core volumes ≥50–70 mL. Third, we conducted sensitivity and subgroup analyses, which confirmed the robustness of our results and accounted for potential biases.

The present meta‐analysis also has several limitations. First, most included studies were observational cohorts rather than RCTs, limiting causal inference. Furthermore, heterogeneity in baseline characteristics, center‐level practices, patient selection, and confounder adjustment across studies may have further biased the pooled results. Second, the limited number of studies reporting comparable adjusted estimates precluded a sensitivity analysis using adjusted ORs. Third, residual confounding and selection bias could not be excluded. The dEVT group likely included both IVT‐eligible and IVT‐ineligible patients, with the latter generally having more comorbidities, anticoagulant use, delayed presentation, or other unfavorable baseline characteristics associated with poorer prognosis. These factors may partially explain the observed superiority of BT and introduce selection bias. Therefore, the results should be interpreted as reflecting the association between BT and clinical outcomes rather than definitive causal evidence. Fourth, substantial clinical heterogeneity exists among the included studies despite low statistical heterogeneity for some outcomes. The studies differed in imaging modality (NCCT vs. DWI‐MRI), EVT time window, thrombolytic protocols, thrombectomy techniques, and study design, which may influence baseline risk, reperfusion rates, hemorrhage risk, and generalizability. For example, patients defined as having large‐core infarction by different imaging modalities may have distinct pathophysiological characteristics. In addition, advances in thrombectomy technology over the study period may affect reperfusion outcomes. Therefore, the pooled results should be interpreted with caution when applying to different clinical settings. Fifth, all pooled estimates were based on unadjusted measures, and because adjusted estimates were not uniform across studies, a sensitivity analysis using adjusted data could not be performed. These limitations should be considered when interpreting the results. Finally, moderate heterogeneity was observed for successful reperfusion, which may reflect differences in study design, treatment time windows, first‐line thrombectomy techniques, and operator experience.

Further large‐scale, high‐quality RCTs are needed to confirm these findings in patients with large‐core ischemic stroke. Future studies should standardize IVT protocols and imaging criteria for defining large infarct cores, incorporate patient‐level analyses to better identify subgroups most likely to benefit from BT, and explore longer‐term outcomes beyond 90 days. The use of advanced imaging techniques, such as perfusion imaging, may also help refine treatment selection and improve individualized decision‐making in this challenging population.

## 5 Conclusion

In conclusion, this meta‐analysis suggests that BT may be associated with improved 90‐day functional outcomes compared with dEVT in patients with large ischemic core infarcts, without a statistically significant increase in the risk of sICH, ICH, or 90‐day mortality. Although no significant differences were observed in SR, the overall findings support a potential functional advantage of BT in this high‐risk population. Nevertheless, because most included data were derived from observational studies, the possibility of residual confounding cannot be excluded. Therefore, large‐scale and high‐quality RCTs are still needed to confirm these findings.

## Author Contributions

B.W. and F.L. performed the literature search, collected the data, conducted the statistical analysis, and drafted the manuscript. G.S. and S.W. designed the study, evaluated the quality of the included studies, and revised the manuscript for intellectual content. All authors have reviewed and approved the final manuscript.

## Funding

The authors have nothing to report.

## Ethics Statement

The authors have nothing to report.

## Consent

The authors have nothing to report.

## Conflicts of Interest

The authors declare no conflicts of interest.

## Supporting information




**Supplementary Materials**: brb371851‐sup‐0001‐SuppMat.docx

## Data Availability

The data used in this study were obtained from published data and are included in this article. For further inquiries, please contact the corresponding author.
