# Supplementary material for: Bridging Thrombolysis Versus Direct Endovascular Thrombectomy in Acute Ischemic Stroke Patients With ASPECTS ≤5: A Meta‐Analysis
Source: Brain Behav. 2026 Oct 22;16(10):e71851. doi: 10.1002/brb3.71851 (PMC13691425; doi:10.1002/brb3.71851)
Supplement: Supplementary file 1 [file BRB3-16-e71851-s001.docx]

Supplemental Figure 1. Forest plot showing the risk difference (RD) for favorable functional outcome (mRS 0–2) at 90 days between BT and dEVT.

Supplemental Figure 2. Forest plot showing the risk difference (RD) for moderate functional outcome (mRS 0–3) at 90 days between BT and dEVT.

Supplemental Figure 3. Forest plot showing the risk difference (RD) for symptomatic intracranial hemorrhage (sICH) between BT and dEVT.

Supplemental Figure 4. Forest plot showing the risk difference (RD) for any intracranial hemorrhage (ICH) between BT and dEVT.

Supplemental Figure 5. Forest plot showing the risk difference (RD) for mortality at 90 days between BT and dEVT.

 Supplemental Figure 6. Forest plot showing the risk difference (RD) for successful reperfusion between BT and dEVT.

Supplemental Figure 7. Forest plot of sensitivity analysis for favorable functional outcome (mRS 0–2) at 90 days.

Supplemental Table 1. PubMed search strategy

| #1 | ((("core"[Title/Abstract] OR "region"[Title/Abstract] OR "infarct"[Title/Abstract] OR "ischemic"[Title/Abstract]) AND "large"[Title/Abstract] )  OR "ASPECT"[Title/Abstract] OR "ASPECTS"[Title/Abstract] OR "alberta stroke program early computed tomography score"[Title/Abstract] OR "alberta stroke program early ct score"[Title/Abstract] OR "alberta stroke program early computed tomographic score"[Title/Abstract]) |
| --- | --- |
| #2 | ("stroke"[MeSH Terms] OR "stroke"[Title/Abstract] OR "brain infarction"[MeSH Terms] OR "brain infarction"[Title/Abstract] OR "cerebral infarction"[MeSH Terms] OR "cerebral infarction"[Title/Abstract] OR "ischemic stroke"[MeSH Terms] OR "ischemic stroke"[Title/Abstract] OR "acute ischemic stroke"[Title/Abstract]) |
| #3 | ("thrombectomy"[MeSH Terms] OR "thrombectomy"[Title/Abstract] OR "mechanical thrombectomy"[Title/Abstract] OR "Endovascular treatment"[Title/Abstract] OR "Endovascular therapy"[Title/Abstract])  AND ("Intravenous thrombolysis"[Title/Abstract] OR "Alteplase"[Title/Abstract] OR "rtPA"[Title/Abstract] OR "Tenecteplase"[Title/Abstract] OR "bridging intravenous thrombolysis"[Title/Abstract] OR "Bridging therapy"[Title/Abstract]) |
| #4 | ("2010/01/01"[Date - Publication] : "2024/09/21"[Date - Publication]) |
| #5 | #1 AND #2 AND #3 AND #4 |

Supplemental Table 2. Quality assessment of enrolled studies using the Newcastle-Ottawa Scale (NOS) for included study
